# Moving Air in the Esophagus During Cryoballoon Ablation

**DOI:** 10.19102/icrm.2019.100807

**Published:** 2019-08-15

**Authors:** Tolga Aksu, Tumer E. Guler, Serdar Bozyel, Kivanc Yalin

**Affiliations:** ^1^Department of Cardiology, University of Health Sciences, Kocaeli Derince Training and Research Hospital, Kocaeli, Turkey; ^2^Department of Cardiology, Uşak University Hospital, Uşak, Turkey

**Keywords:** Atrial fibrillation, gastroparesis, parasympathetic, vagus

## Abstract

A 56-year-old male patient underwent cryoballoon ablation for symptomatic paroxysmal atrial fibrillation. Massive air movement reminiscent of an air esophagram was detected during cryoballoon application in the right superior pulmonary vein. In this case report, we sought to consider all possible explanations of this finding.

## Case presentation

**Figure 1:**
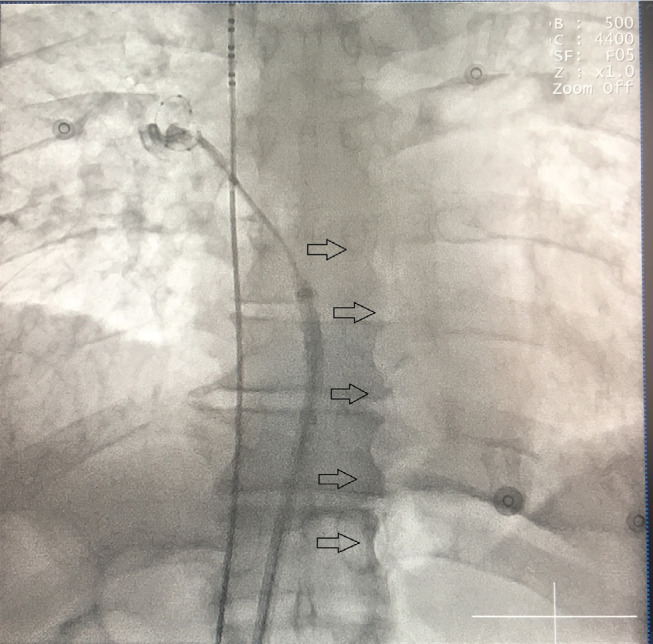
Cryoballoon catheter and decapolar catheter located in the right phrenic nerve and the superior vena cava, respectively.

A 56-year-old male patient underwent cryoballoon ablation for symptomatic paroxysmal atrial fibrillation under local anesthesia and moderate sedation with midazolam. Although isolation of the left pulmonary veins (PVs) was successfully completed without acute complications, massive air movement reminiscent of an air esophagram was detected during cryoballoon application in the right superior PV **(Figure 1 and Video 1)**. The patient raised no complaint of discomfort and his vital signs including blood pressure, heart and respiratory rates, and oxygen saturation were all within normal ranges.

To reveal the mechanism, the procedure was halted and fluoroscopy views were checked in detail. One of the possible explanations was cryoballoon burst and fistulization of the air from the balloon into the esophagus. However, there was no change in the balloon size observed during application. The esophagus was also located far from the right superior PV. This may be accepted as a finding that excludes another possible complication, esophageal fistula. Furthermore, air moves from the esophagus to the left atrium, not the other way. Ultimately, the patient was diagnosed with excessive air swallowing.

## Discussion

**Figure 2:**
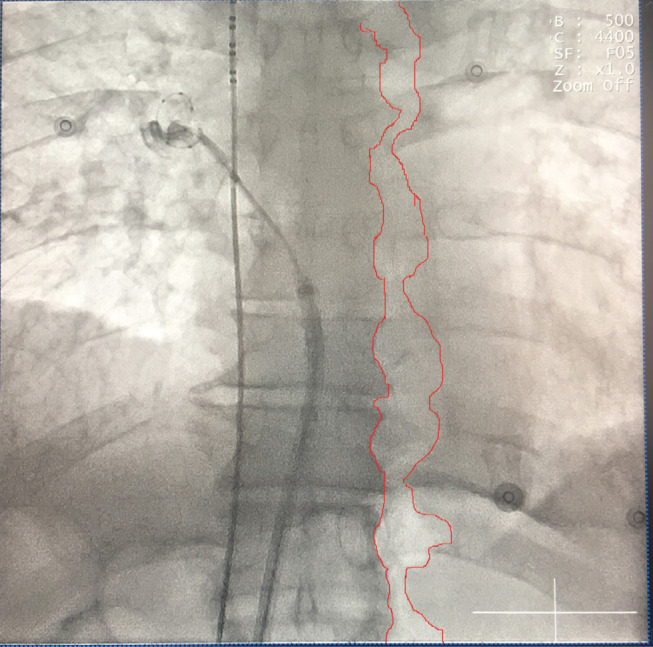
The same scopic view as seen in **Figure 1**. The border of the esophagus is marked with a red line to facilitate demonstration of the esophagus.

Gastroparesis is a syndrome characterized by delayed gastric emptying in the absence of mechanical obstruction of the stomach.^1^ The disorder is associated with symptoms such as epigastric discomfort, abdominal pain, nausea, vomiting, and bloating and can be caused by periesophageal vagal nerve injury during cryoballoon ablation.^2^ The diagnosis may be confirmed by fluoroscopy upon viewing an air-filled stomach or reviewing the air–fluid level in the fundus of an enlarged fluid-filled stomach during cryoablation.^2^ If the movement of the air is carefully examined, it can sometimes be observed that an air bubble is arising from the mouth, as was true in this case **(Figure 2 and Video 1)**. Excessive or repetitive air swallowing is called aerophagia.^3^ Excess swallowed air distends the stomach and initiates transient lower esophageal sphincter relaxation. With this occurrence, air enters into and distends the esophagus, an event that induces reflex relaxation of the upper esophageal sphincter, and air is vented through the mouth. Although the observation of air within the esophagus during chest radiography has almost always been reported in association with other detectable pulmonary parenchymal or mediastinal abnormalities, it may also demonstrate just an aerophagia, such as was true in the current case.^4^

The reason for the excessive or repetitive air-swallowing remains uncertain. The patient had no clinical history of symptoms related to aerophagia. In our case, one of the reasons for the phenomenon may be that it is a response to the perception of an unpleasant pain stimulus during cryoballoon application, as described in patients with reflux symptoms.^5^ Stimulation of the parasympathetic system elicits a complete swallowing reflex, including pharyngeal and esophageal peristalsis and lower esophageal sphincter relaxation.^6^ Cryoballoon application may promote vagal discharge due to the close spatial relationship between the left atrial ganglionated plexi and the PVs.^7^ Vagal response may be the other possible explanation of aerophagia in the present case.

To the best of our knowledge, there is no controlled trial in existence that has investigated the treatment of aerophagia. Therefore, management suggestions are based mainly on expert opinions. In patients with acute and severe episodes of aerophagia, a nasogastric tube to relieve gastric air seems reasonable and sedatives such as lorazepam may help to reduce repetitive air-swallowing.^8^ In the present case, we continued the procedure without the application of a special treatment because the patient was totally asymptomatic.

In conclusion, aerophagia during cryoballoon application for PV isolation may be a transient and completely reversible phenomenon. The eliciting of a complete swallowing reflex caused by excessive vagal discharge through neurally mediated pathways appears to be a possible explanation for this unexpected complication. However, other possible and potentially devastating complications should always be considered in these cases.

## Figures and Tables

**Video 1:** Moving air in the esophagus.
